# COMT-by-Sex Interaction Effect on Psychosis Proneness

**DOI:** 10.1155/2015/829237

**Published:** 2015-02-05

**Authors:** Marta de Castro-Catala, Neus Barrantes-Vidal, Tamara Sheinbaum, Artal Moreno-Fortuny, Thomas R. Kwapil, Araceli Rosa

**Affiliations:** ^1^Unitat d'Antropologia, Departament de Biologia Animal, Facultat de Biologia, Universitat de Barcelona (UB), Avinguda Diagonal 643, 08028 Barcelona, Spain; ^2^Departament de Psicologia Clínica i de la Salut, Facultat de Psicologia, Universitat Autònoma de Barcelona (UAB), Bellaterra, 08193 Barcelona, Spain; ^3^Department of Psychology, University of North Carolina at Greensboro, Greensboro, NC 27402-6170, USA; ^4^Sant Pere Claver-Fundació Sanitària, Carrer Vila i Vilà 16, 08004 Barcelona, Spain; ^5^Centre for Biomedical Research Network on Mental Health (CIBERSAM), Instituto de Salud Carlos III, 28029 Madrid, Spain; ^6^Institute of Inflammation and Repair, University of Manchester, Oxford Road, Manchester M13 9PL, UK

## Abstract

Schizotypy phenotypes in the general population share etiopathogenic mechanisms and risk factors with schizophrenia, supporting the notion of psychosis as a continuum ranging from nonclinical to clinical deviance. Catechol-O-methyltransferase (COMT) is a candidate susceptibility gene for schizophrenia that is involved in the regulation of dopamine in the prefrontal cortex. Several recent studies have reported a sex difference in the impact of COMT genotype on psychiatric and cognitive phenotypes and personality traits. The present study investigated the association of COMT Val158Met (rs4680) with psychometric positive and negative schizotypy and psychotic experiences in a sample of 808 nonclinical young adults. The main finding was that sex moderates the association of COMT genotype with the negative dimension of both schizotypy and psychotic experiences. Male subjects carrying the Val allele tended to score higher on the negative dimension of both trait and symptom-like measures. The results from the present study are consistent with recent work suggesting an association between negative schizotypy and diminished prefrontal dopamine availability. They support the idea that a biological differentiation underlies the positive and negative schizotypy dimensions. Additionally, these findings contribute to the growing literature on sex-specific effects of COMT on the predisposition to psychiatric disorders and personality traits.

## 1. Introduction

Functional, clinical, and genetic epidemiological studies show that many parameters of brain function and structure vary between men and women [1]. Similarly, most psychiatric disorders show sex differences in characteristics such as incidence, age at onset, clinical features, and outcome [2]. These differences are usually ascribed to the influence of sex hormones or the action of sex chromosome genes. However, there is evidence that autosomal genes may contribute to sex differences in the genetic predisposition to psychiatric phenotypes [3]. Despite this, the identity of the contributing genes is largely unknown. Several lines of evidence suggest that the catechol-O-methyltransferase (COMT) gene, which codes for an enzyme that plays an important role in the cortical dopamine metabolism, may be one such gene. It contains a functional polymorphism, a G>A substitution (rs4680), which produces a valine-to-methionine (Val/Met) substitution that influences COMT activity in the human prefrontal cortex. Thus, dopamine signalling is likely to be enhanced in Met carriers as compared to Val carriers. The deficit and excess of dopamine have been related to positive and negative schizophrenia symptoms [4]. It has been proposed that positive symptoms are due to a hyperactivity of subcortical dopamine transmission, while negative symptoms may be caused by a deficit in dopamine transmission, particularly in the prefrontal cortex [5, 6]. However, the precise mechanisms are still not well understood.

Several studies have reported a sex difference in the impact of COMT genotype on psychiatric phenotypes and personality traits (reviewed by Harrison and Tunbridge [7, 8]), but few have explored the hypothesis of an interaction between sex and genotype [9, 10].

The association between schizophrenia and the high activity Val allele has been analysed in several studies that have culminated in different meta-analysis which showed inconsistent results [11, 12]. The influence of sex on the involvement of COMT genotype in schizophrenia vulnerability has been reported in several studies [13–16]. Candidate endophenotypes of schizophrenia, with which the disorder presumably shares a degree of overlapping genetic liability, include structural and morphometric brain alterations, neurocognitive deficits, and schizotypal personality traits or symptoms.

The underlying developmental vulnerability for schizophrenia and spectrum disorders is expressed across a dynamic continuum referred to as schizotypy or “psychosis proneness” that ranges from subclinical to clinical manifestations [17–19]. Nonpsychotic schizotypes experience similar, although attenuated, forms of the cognitive, emotional, and behavioural disturbances inherent in schizophrenia and are at heightened risk for developing schizophrenia-spectrum disorders [20]. Like schizophrenia, schizotypy is a multidimensional construct, positive and negative schizotypy dimensions being the most replicated factors [21]. According to family, twin and adoption studies [22] and prior genome-wide scans of schizophrenia and schizotypy, it seems that at least a subset of schizophrenia susceptibility genes also affect schizotypy in nonpsychotic relatives [23].

The nonclinical psychosis phenotype is observed and reliably measured at the level of schizotypy personality features (using trait-like measures) and subclinical psychotic experiences (PEs, i.e., unusual phenomena resembling clinical psychotic experiences) (using symptom-based measures). These phenotypes seem to share etiopathogenic mechanisms and risk factors with schizophrenia, thus supporting the notion of psychosis as a continuum ranging from nonclinical to clinical deviance [21, 24–28].

Evidence that the COMT Val158Met polymorphism may indeed have an impact on schizotypal endophenotypes was provided by Avramopoulos and colleagues [29]. They reported that the high activity Val allele is associated with self-reported schizotypy scores in a male population using the Perceptual Aberration Scale and the total score of the Schizotypal Personality Questionnaire. Further work showed that the Val allele was specifically related to the negative and disorganised dimensions of schizotypy [30, 31]. More recently, two independent studies analysing subjects carrying genetic liability for schizophrenia have found associations between the high activity allele and positive and negative schizotypy [32] and anhedonia (a central construct of negative schizotypy) [33]. To our knowledge, the association between COMT variability and PEs has not been analysed previously.

The present study aimed to explore (i) the association between this functional polymorphism in the COMT gene and dimensions of psychosis proneness using trait-like and symptom-based measures and (ii) sex-specific effects of the COMT on this phenotype in a sample of nonclinical young adults. We hypothesised that the Val allele of the Val158Met polymorphism, associated with diminished prefrontal dopamine availability, would be associated with higher scores on the negative dimension of both schizotypy and PEs.

## 2. Materials and Methods

### 2.1. Participants

The sample comprised 808 subjects from the general population, including 547 undergraduates enrolled in psychology courses at the Universitat Autònoma de Barcelona (UAB) and 261 students from technical training schools from Barcelona. Details of the two subsamples are given in Table 1. The final sample comprised 184 men (23%) and 624 women (77%). Males and females differed in terms of age (males: mean = 21.4, SD = 4.5; females: mean = 20.6, SD = 3.9, *P* < 0.05). Ethical approval was obtained from local research ethics committees. All participants volunteered to take part in the study and provided written informed consent. They were not preselected based upon any criteria.

### 2.2. Psychosis Proneness Assessment

All participants completed self-report measures assessing schizotypy and PEs. Schizotypy was assessed with the Spanish version [34] of the Wisconsin Schizotypy Scales (WSS), including the Perceptual Aberration [35], Magical Ideation [36], Revised Social Anhedonia [37], and Physical Anhedonia [38] Scales. The technical school volunteers completed the short version of the scales [39]. Exploratory and confirmatory factor analyses of the four scales reliably produce two factors, positive and negative schizotypy, that account for 80% of the variance [21]. Although the raw scores on the four scales are not comparable between the two samples, the factor structure underlying the short scales is comparable with the factor structure of the original scales (data not shown). Participants were assigned positive and negative schizotypy factor scores based upon factor loadings derived from a sample of 6137 college students [21].

PEs were assessed with the Spanish version of the Community Assessment of Psychic Experiences (CAPE) [34, 40], which evaluates three dimensions of symptoms: positive, negative, and depressive (the depressive subscale was not used in the present study). It has good validity and reliability and has been used in general population studies [41].

### 2.3. Genotyping

Genomic DNA was extracted from buccal mucosa on a cotton swab using the Real Extraction DNA kit (Durviz S.L.U., Valencia, Spain). DNA quality from all the samples was assessed by spectrophotometer readings (A260/280) using Nanodrop.

The COMT Val158Met polymorphism (rs4680, G>A) was genotyped using TaqMan 5′ exonuclease assay (Applied Biosystems). The probe for genotyping the rs4680 was ordered through the TaqMan SNP genotyping Assays (ID: C_25746809_50) Applied Biosystems assay-on-demand service. The final volume was 5 *μ*L, which contained 5 ng of genomic DNA, 2.5 *μ*L of TaqMan Master Mix, and 0.25 *μ*L of 20 genotyping assay. Polymerase chain reaction plates were read on an ABI PRISM 7900HT instrument and SDS v2.1 software (Applied Biosystems) was used for the genotype analysis of data.

### 2.4. Statistical Analyses

Hardy-Weinberg equilibrium for genotypic distribution was analysed using an online chi-squared Hardy-Weinberg equilibrium test calculator for biallelic markers (http://www.oege.org/software/hwe-mr-calc.shtml) [42].

All data were processed using SPSS 20.0 software. ANCOVA (analysis of covariance) was performed to test the effect of COMT Val158Met genotype (Val/Val, Val/Met, and Met/Met) on the positive and negative schizotypy and PEs scores. We also used ANCOVA to study the interaction between sex and COMT genotype on positive and negative schizotypy and PEs. The analyses were repeated comparing subjects who were carriers of at least one Val allele (Val carriers: Val/Val and Val/Met genotypes) versus Met/Met subjects.

## 3. Results

The total sample comprised 808 subjects. Differences were observed between the undergraduate and the technical school students in relation to age and sex. Also, the positive and negative schizotypy scores and the positive PEs scores were higher in the technical school sample than in the undergraduate student sample. See Table 1 for more details on the two groups. All the analyses were corrected by sex, age, and also considering whether individuals were from the university student sample or from the technical training school sample.

Of the whole sample, 805 individuals agreed on providing a DNA sample. COMT genotyping failed for 31 individuals. The two alleles Val and Met were present at overall frequencies of 52.8% and 47.2%, respectively. The three genotypes were Val/Val 28.7% (223/777), Val/Met 48.1% (374/777), and Met/Met 23.2% (180/777). The genotypes were in Hardy-Weinberg equilibrium. Both genotypic and allelic frequencies observed in our sample are in accordance with those found in other European populations and previous studies [30]. Mean scores and standard deviations of the WSS factors and CAPE subscales for the whole sample, as well as by genotype, are shown in Table 2.

The association of positive and negative schizotypy with the Val158Met genotype was not significant (positive schizotypy: *F* = 0.244,  *P* = 0.783; negative schizotypy: *F* = 0.634,  *P* = 0.531; see Table 2). The lack of association held when analyses were conducted in the sample of males and females independently (data not shown). Two-way ANCOVA analyses (genotype∗sex) were conducted separately for positive and negative schizotypy. A significant interaction was found between COMT genotypes (Val/Val, Val/Met, and Met/Met genotypes) and sex with regard to negative schizotypy (*F*_(2,772)_ = 3.924,  *P* = 0.020). No significant interaction was found for positive schizotypy (*F*_(2,772)_ = 0.232,  *P* = 0.793).

The association between the two subscales of the CAPE and the Val158Met genotype was not significant (positive PEs: *F* = 1.485,  *P* = 0.227; negative PEs: *F* = 0.508,  *P* = 0.602; see Table 2). When analyses were conducted in males and females independently, a significant association was found in males with regard to negative PEs. Specifically, subjects with the Val/Met genotype showed higher scores than Val/Val and Met/Met subjects (*F* = 3.591,  *P* = 0.030). Two-way ANCOVA analyses (genotype∗sex) were conducted separately for positive and negative PEs. A significant interaction was detected between the three COMT genotypes and sex with regard to negative PEs (*F*_(2,772)_ = 4.229,  *P* = 0.015). No significant interaction was detected with positive PEs (*F*_(2,775)_ = 0.152,  *P* = 0.859).

**Figure 1:**
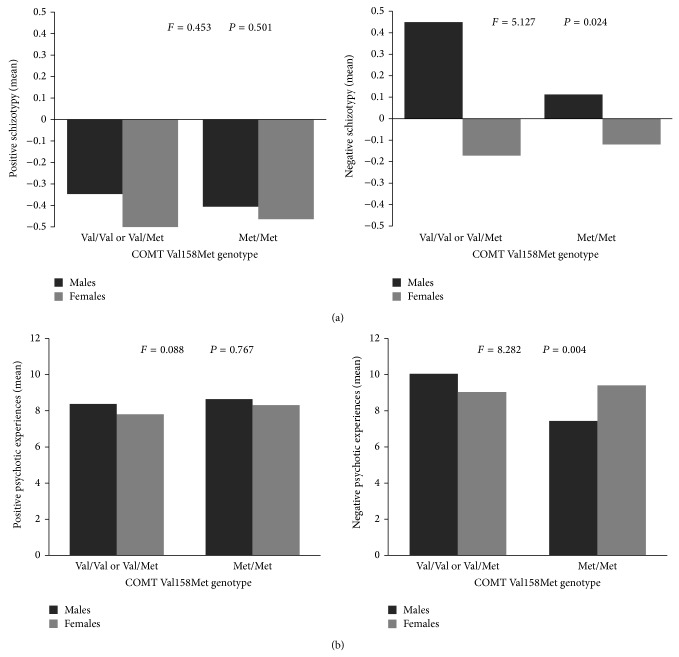
Mean scores of the psychosis proneness variables by sex and genotype (Val carriers versus Met/Met). (a) Positive and negative schizotypy and (b) positive and negative psychotic experiences.

Further analyses grouping the COMT genotypes in Val carriers (Val/Val and Val/Met) and Met/Met revealed that these significant interactions resulted mostly from significant effects of the COMT Val allele in males. Males carrying Val alleles showed significantly higher scores for the negative dimension of both schizotypy (negative schizotypy: *F*_(1,772)_ = 5.127, *P* = 0.024; see Figure 1(a)) and PEs (negative PEs: *F*_(1,772)_ = 8.282, *P* = 0.004; see Figure 1(b)). Females, in contrast, did not show this pattern (Figure 1).

## 4. Discussion

In the current study we tested the hypothesis that the functional polymorphism Val158Met in the COMT gene would be associated with the negative dimension of the psychosis proneness phenotype in a nonclinical sample. Such phenotypes often referred to as “intermediate phenotypes” or endophenotypes with which schizophrenia presumably shares a degree of overlapping genetic liability are useful in studying the potential effect of candidate susceptibility genes for the disorder. Our main finding is that sex modulated the association between the COMT genotype and the negative dimension of both schizotypy and PEs.

These findings contribute to the growing literature on sex-specific effects of the role of COMT in the vulnerability to psychiatric or cognitive phenotypes and personality traits [10, 43–45]. In the present study, male subjects carrying the Val allele tended to score higher on the negative dimension of both trait and symptom-like measures. Our results are consistent with several studies that have investigated, at the population level, schizotypal traits as phenotypes in relation to the COMT in samples of young men [29–31]. In other nonclinical studies, although hypotheses regarding sex-specific associations were not specifically addressed, a trend towards a specific association in males was also detected [32, 46]. The association between COMT and CAPE scores has only been examined in studies of gene-environment interaction. In this regard, recent studies have shown that COMT constitutes a genetic risk factor for PEs in the context of combined exposure to childhood maltreatment and/or cannabis use [47–50]. However, the neurobiological bases of this interaction remain poorly understood.

The role of COMT in schizophrenia has been extensively studied and it seems that neither genetic variants nor the catalytic activity of the enzyme has great intrinsic influence on schizophrenia risk. However, multiple lines of evidence indicate that the high-activity COMT Val allele is associated with greater severity of negative and cognitive symptoms in schizophrenia, as well as specific endophenotypic impairments related to prefrontal deficits such as schizotypal traits [51]. In this regard, neuroimaging studies seem to point out the importance of low dopaminergic activity in the prefrontal cortex of schizophrenia patients. Specifically, the COMT Val carriers, with high enzyme activity, may have reduced dopamine levels in several brain regions and specifically in the prefrontal cortex, leading to a decrease in D1 receptors activation with subsequent impairment in cognitive tasks, such as working memory, as described in patients and healthy individuals [52]. The neurobiological mechanism underlying this association is highly debated and emerging findings from animal models seem to suggest that the significance of COMT for dopamine regulation is not limited to the prefrontal cortex [53].

The sex-specific association of the COMT gene with the negative dimension of psychosis proneness described here is in line with several studies in schizophrenia (reviewed by Godar and Bortolato [54]). In this regard, it seems that the effect of the Val158Met polymorphism on schizophrenia vulnerability is more directly related to male patients, possibly through an epistatic interaction with D1 receptors [15].

Higher COMT activity in the prefrontal cortex in men occurs despite a similar expression of the genes (i.e., mRNA and proteins) in both sexes. This dissociation between expression and activity may be explained by the ability of testosterone to increase COMT activity or the effect of estrogens. In this respect, it seems that there is a reciprocal COMT-estrogen modulation by which the COMT genotype influences the role that estrogens play in the brain, while estrogens affect COMT activity and its pathophysiological phenotype. An additional mechanism that may predict a lower COMT activity in females may be afforded by the function of catechol-estrogens and its role modulating the turnover of catecholamines. Further evidence on sexual dimorphic effects of this gene is provided by COMT knockout mouse studies. They provide evidence for an important sex- and region-specific contribution of COMT in the maintenance of steady-state levels of catecholamines in the brain and suggest a role for COMT in some aspects of emotional and social behaviour in mice [55].

The finding of an association between the COMT polymorphism and the negative dimension of the psychosis proneness phenotype provides evidence that the biological effects of the COMT gene may be relevant to the pathophysiology of schizophrenia. The balance of dopamine in prefrontal and striatal regions might be related to schizotypal characteristics evident in individuals who carry genetic liability for the condition. COMT gene is only a part of the complex neural dopaminergic system. Other dopamine genes and receptors and possibly other systems such as the serotoninergic system may all interact with the effects of this gene. The current evidence on the implication of other dopaminergic targets in gene-sex interactions is scant and mostly limited to dopaminergic receptors. Interestingly, several studies have shown that polymorphic variants in D1, D2, and D4 receptors are linked to different responses to antipsychotic medication in a gender-sensitive fashion.

The findings of this and previous studies seem to indicate that the role of gene∗sex interactions might affect brain substrates through different mechanisms with a particular impact on the catabolic action on dopamine. A comprehensive understanding of the genetic basis of schizotypy needs to consider the contributions of multiple genes and also environmental and biological factors. In this regard, the integration of preclinical research with neuroimaging and genetic studies will play a critical role enabling us to identify central neurobiological networks that underpin sex-specific, neurobehavioral endophenotypes of schizophrenia.

## Figures and Tables

**Table 1:** Descriptive data for the undergraduate and technical training school students and comparison between the two subsamples.

	Undergraduates	Technical school	
Age (mean (SD))	20.60 (4.1)	21.20 (3.9)	*t* = −2.01*P* = **0.045**
Sex (men/women)	92/455	92/169	*χ* ^2^ = 34.13*P* = **0.000**
COMT Val158Met (*n* (%))			
Val/Val	151 (28.8%)	72 (28.5%)	
Val/Met	253 (48.3%)	121 (47.8%)	*χ* ^2^ = 0.064*P* = 0.969
Met/Met	120 (22.9%)	60 (23.7%)	
Schizotypy (WSS) (mean (SD))			
Positive	−0.55 (0.8)	−0.26 (0.7)	*t* = −5.33*P* = **0.000**
Negative	−0.18 (0.9)	0.24 (0.9)	*t* = −6.19*P* = **0.000**
Psychotic experiences (CAPE) (mean (SD))			
Positive	7.24 (4.3)	9.72 (5.5)	*t* = −6.96*P* = **0.000**
Negative	8.92 (5.1)	9.62 (5.6)	*t* = −1.69*P* = 0.092

WSS, Wisconsin Schizotypy Scales; CAPE, Community Assessment of Psychic Experiences. Differences were considered significant if *P* was below 0.05 (shown in bold).

**Table 2:** Descriptive data and ANCOVA statistics for the two WSS factors and CAPE subscales in relation to the COMT Val158Met genotype.

	Total sample mean (SD)	Val/Val mean (SD)	Val/Met mean (SD)	Met/Met mean (SD)	*F* (*P*)
Schizotypy (WSS)					
Positive	−0.46 (0.75)	−0.44 (0.85)	−0.48 (0.70)	−0.45 (0.74)	0.244 (0.783)
Negative	−0.04 (0.90)	−0.06 (0.90)	−0.01 (0.90)	−0.06 (0.90)	0.634 (0.531)
Psychotic experiences (CAPE)					
Positive	8.05 (4.85)	8.18 (4.84)	7.80 (4.78)	8.39 (5.01)	1.485 (0.227)
Negative	9.19 (5.26)	9.06 (4.96)	9.40 (5.45)	8.93 (5.24)	0.508 (0.602)

WSS, Wisconsin Schizotypy Scales; CAPE, Community Assessment of Psychic Experiences. Differences were considered significant if *P* was below 0.05.

## References

[1] Cosgrove K. P., Mazure C. M., Staley J. K. (2007). Evolving knowledge of sex differences in brain structure, function, and chemistry. *Biological Psychiatry*.

[2] Aleman A., Kahn R. S., Selten J.-P. (2003). Sex differences in the risk of schizophrenia: evidence from meta-analysis. *Archives of General Psychiatry*.

[3] Cahill L. (2006). Why sex matters for neuroscience. *Nature Reviews Neuroscience*.

[4] Davis K. L., Kahn R. S., Ko G., Davidson M. (1991). Dopamine in schizophrenia: a review and reconceptualization. *The American Journal of Psychiatry*.

[5] Wang Y., Fang Y., Shen Y., Xu Q. (2010). Analysis of association between the catechol-O-methyltransferase (COMT) gene and negative symptoms in chronic schizophrenia. *Psychiatry Research*.

[6] Laruelle M. (2014). Schizophrenia: from dopaminergic to glutamatergic interventions. *Current Opinion in Pharmacology*.

[7] Harrison P. J., Tunbridge E. M. (2008). Catechol-O-methyltransferase (COMT): a gene contributing to sex differences in brain function, and to sexual dimorphism in the predisposition to psychiatric disorders. *Neuropsychopharmacology*.

[8] Tunbridge E. M., Harrison P. J. (2011). Importance of the COMT gene for sex differences in brain function and predisposition to psychiatric disorders. *Current Topics in Behavioral Neurosciences*.

[9] Nolan K. A., Volavka J., Czobor P. (2000). Suicidal behavior in patients with schizophrenia is related to COMT polymorphism. *Psychiatric Genetics*.

[10] Lang U. E., Bajbouj M., Sander T., Gallinat J. (2007). Gender-dependent association of the functional catechol-O-methyltransferase Val158Met genotype with sensation seeking personality trait. *Neuropsychopharmacology*.

[11] Munafò M. R., Bowes L., Clark T. G., Flint J. (2005). Lack of association of the COMT (Val158/108 Met) gene and schizophrenia: a meta-analysis of case-control studies. *Molecular Psychiatry*.

[12] Costas J., Sanjuán J., Ramos-Ríos R. (2011). Heterozygosity at catechol-O-methyltransferase Val158Met and schizophrenia: new data and meta-analysis. *Journal of Psychiatric Research*.

[13] Shifman S., Bronstein M., Sternfeld M. (2002). A highly significant association between a COMT haplotype and schizophrenia. *The American Journal of Human Genetics*.

[14] Sazci A., Ergul E., Kucukali I., Kilic G., Kaya G., Kara I. (2004). Catechol-O-methyltransferase gene Val108/158Met polymorphism, and susceptibility to schizophrenia: association is more significant in women. *Molecular Brain Research*.

[15] Hoenicka J., Garrido E., Ponce G. (2010). Sexually dimorphic interaction between the DRD1 and COMT genes in schizophrenia. *American Journal of Medical Genetics, Part B: Neuropsychiatric Genetics*.

[16] Hoenicka J., Garrido E., Martínez I. (2010). Gender-specific COMT Val158Met polymorphism association in Spanish schizophrenic patients. *The American Journal of Medical Genetics. Part B: Neuropsychiatric Genetics*.

[17] Claridge G., McCreery C., Mason O. (1996). The factor structure of ‘schizotypal’ traits: a large replication study. *British Journal of Clinical Psychology*.

[18] Meehl P. E. (1990). Toward an integrated theory of schizotaxia, schizotypy, and schizophrenia. *Journal of Personality Disorders*.

[19] Kwapil T., Barrantes-Vidal N. (2012). Schizotypal personality disorder: an integrative review. *The Oxford Handbook of Personality Disorders*.

[20] Barrantes-Vidal N., Gross G. M., Sheinbaum T., Mitjavila M., Ballespí S., Kwapil T. R. (2013). Positive and negative schizotypy are associated with prodromal and schizophrenia-spectrum symptoms. *Schizophrenia Research*.

[21] Kwapil T. R., Barrantes-Vidal N., Silvia P. J. (2008). The dimensional structure of the wisconsin schizotypy scales: factor identification and construct validity. *Schizophrenia Bulletin*.

[22] Kendler K. S., Thacker L., Walsh D. (1996). Self-report measures of schizotypy as indices of familial vulnerability to schizophrenia. *Schizophrenia Bulletin*.

[23] Fanous A. H., Neale M. C., Gardner C. O. (2007). Significant correlation in linkage signals from genome-wide scans of schizophrenia and schizotypy. *Molecular Psychiatry*.

[24] Stefanis N. C., Hanssen M., Smirnis N. K. (2002). Evidence that three dimensions of psychosis have a distribution in the general population. *Psychological Medicine*.

[25] Kwapil T. R., Gross G. M., Silvia P. J., Barrantes-Vidal N. (2013). Prediction of psychopathology and functional impairment by positive and negative schizotypy in the chapmans’ ten-year longitudinal study. *Journal of Abnormal Psychology*.

[26] Barrantes-Vidal N., Lewandowski K. E., Kwapil T. R. (2010). Psychopathology, social adjustment and personality correlates of schizotypy clusters in a large nonclinical sample. *Schizophrenia Research*.

[27] Barrantes-Vidal N., Chun C. A., Myin-Germeys I., Kwapil T. R. (2013). Psychometric schizotypy predicts psychotic-like, paranoid, and negative symptoms in daily life. *Journal of Abnormal Psychology*.

[28] van Os J., Linscott R. J., Myin-Germeys I., Delespaul P., Krabbendam L. (2009). A systematic review and meta-analysis of the psychosis continuum: evidence for a psychosis proneness-persistence-impairment model of psychotic disorder. *Psychological Medicine*.

[29] Avramopoulos D., Stefanis N. C., Hantoumi I., Smyrnis N., Evdokimidis I., Stefanis C. N. (2002). Higher scores of self reported schizotypy in healthy young males carrying the COMT high activity allele. *Molecular Psychiatry*.

[30] Stefanis N. C., van Os J., Avramopoulos D. (2004). Variation in catechol-O-methyltransferase val 158 met genotype associated with schizotypy but not cognition: a population study in 543 young men. *Biological Psychiatry*.

[31] Smyrnis N., Avramopoulos D., Evdokimidis I., Stefanis C. N., Tsekou H., Stefanis N. C. (2007). Effect of schizotypy on cognitive performance and its tuning by COMT val158 met genotype variations in a large population of young men. *Biological Psychiatry*.

[32] Schürhoff F., Szöke A., Chevalier F. (2007). Schizotypal dimensions: an intermediate phenotype associated with the COMT high activity allele. *The American Journal of Medical Genetics, Part B: Neuropsychiatric Genetics*.

[33] Docherty A. R., Sponheim S. R. (2008). Anhedonia as a phenotype for the Val158Met COMT polymorphism in relatives of schizophrenia patients. *Journal of Abnormal Psychology*.

[34] Ros-Morente A., Rodriguez-Hansen G., Vilagrá-Ruiz R., Kwapil T. R., Barrantes-Vidal N. (2010). Adaptation of the wisconsin scales of psychosis proneness to Spanish. *Actas españolas de psiquiatría*.

[35] Chapman L. J., Chapman J. P., Raulin M. L. (1978). Body-image aberration in schizophrenia. *Journal of Abnormal Psychology*.

[36] Eckblad M., Chapman L. J. (1983). Magical ideation as an indicator of schizotypy. *Journal of Consulting and Clinical Psychology*.

[37] Eckblad M., Chapman L., Chapman J., Mishlove M. (1982). *The Revised Social Anhedonia Scale (unpublished test copies)*.

[38] Chapman L. J., Chapman J. P., Raulin M. L. (1976). Scales for physical and social anhedonia. *Journal of Abnormal Psychology*.

[39] Winterstein B. P., Silvia P. J., Kwapil T. R., Kaufman J. C., Reiter-Palmon R., Wigert B. (2011). Brief assessment of schizotypy: developing short forms of the Wisconsin Schizotypy Scales. *Personality and Individual Differences*.

[40] Ros-Morente A., Vilagrà-Ruiz R., Rodríguez-Hansen G., Wigman J., Barrantes-Vidal N. (2011). Proceso de adaptación al castellano de la Escala de Evaluación Comunitaria de Experiencias Psíquicas (CAPE). *Actas Españolas Psiquiatr*.

[41] Konings M., Bak M., Hanssen M., van Os J., Krabbendam L. (2006). Validity and reliability of the CAPE: a self-report instrument for the measurement of psychotic experiences in the general population. *Acta Psychiatrica Scandinavica*.

[42] Rodriguez S., Gaunt T. R., Day I. N. M. (2009). Hardy-Weinberg equilibrium testing of biological ascertainment for Mendelian randomization studies. *The American Journal of Epidemiology*.

[43] Chen C., Chen C., Moyzis R. (2011). Sex modulates the associations between the COMT gene and personality traits. *Neuropsychopharmacology*.

[44] Amstadter A. B., MacPherson L., Wang F. (2012). The relationship between risk-taking propensity and the *COMT* Val^158^Met polymorphism among early adolescents as a function of sex. *Journal of Psychiatric Research*.

[45] Goldberg X., Fatjó-Vilas M., Alemany S., Nenadic I., Gastó C., Fañanás L. (2013). Gene-environment interaction on cognition: a twin study of childhood maltreatment and COMT variability. *Journal of Psychiatric Research*.

[46] Ma X., Sun J., Yao J. (2007). A quantitative association study between schizotypal traits and COMT, PRODH and BDNF genes in a healthy Chinese population. *Psychiatry Research*.

[47] Henquet C., Rosa A., Krabbendam L. (2006). An experimental study of catechol-O-methyltransferase Val158Met moderation of Δ-9-tetrahydrocannabinol-induced effects on psychosis and cognition. *Neuropsychopharmacology*.

[48] Alemany S., Arias B., Fatjó-Vilas M. (2014). Psychosis-inducing effects of cannabis are related to both childhood abuse and COMT genotypes. *Acta Psychiatrica Scandinavica*.

[49] Ramsay H., Kelleher I., Flannery P. (2013). Relationship between the COMT-Val158Met and BDNF-Val66Met polymorphisms, childhood trauma and psychotic experiences in an adolescent general population sample. *PLoS ONE*.

[50] Vinkers C. H., van Gastel W. A., Schubart C. D. (2013). The effect of childhood maltreatment and cannabis use on adult psychotic symptoms is modified by the COMT Val^158^Met polymorphism. *Schizophrenia Research*.

[51] Egan M. F., Goldberg T. E., Kolachana B. S. (2001). Effect of COMT Val108/158 Met genotype on frontal lobe function and risk for schizophrenia. *Proceedings of the National Academy of Sciences of the United States of America*.

[52] Rosa A., Peralta V., Cuesta M. J. (2004). New evidence of association between COMT gene and prefrontal neurocognitive function in healthy individuals from sibling pairs discordant for psychosis. *American Journal of Psychiatry*.

[53] Laatikainen L. M., Sharp T., Harrison P. J., Tunbridge E. M. (2013). Sexually dimorphic effects of catechol-O-methyltransferase (COMT) inhibition on dopamine metabolism in multiple brain regions. *PLoS ONE*.

[54] Godar S. C., Bortolato M. (2014). Gene-sex interactions in schizophrenia: focus on dopamine neurotransmission. *Frontiers in Behavioral Neuroscience*.

[55] Gogos J. A., Morgan M., Luine V. (1998). Catechol-O-methyltransferase-deficient mice exhibit sexually dimorphic changes in catecholamine levels and behavior. *Proceedings of the National Academy of Sciences of the United States of America*.

